# The potential use of cuticular hydrocarbons and multivariate analysis to age empty puparial cases of *Calliphora vicina* and *Lucilia sericata*

**DOI:** 10.1038/s41598-017-01667-7

**Published:** 2017-05-16

**Authors:** Hannah E. Moore, Jennifer L. Pechal, M. Eric Benbow, Falko P. Drijfhout

**Affiliations:** 1 Cranfield Forensic Institute, Cranfield University, Shrivenham, Swindon, SN6 8LA UK; 2 Department of Entomology, 243 Natural Science, Michigan State University, East Lansing, MI USA; 3 Department of Osteopathic Medical Specialties, 243 Natural Science, Michigan State University, East Lansing, MI USA; 4 Ecology, Evolutionary Biology, and Behavior Program, 243 Natural Science, Michigan State University, East Lansing, MI USA; 5 Department of Chemical Ecology, School of Chemical and Physical Science, Keele University, ST5 5BG England, UK

## Abstract

Cuticular hydrocarbons (CHC) have been successfully used in the field of forensic entomology for identifying and ageing forensically important blowfly species, primarily in the larval stages. However in older scenes where all other entomological evidence is no longer present, Calliphoridae puparial cases can often be all that remains and therefore being able to establish the age could give an indication of the PMI. This paper examined the CHCs present in the lipid wax layer of insects, to determine the age of the cases over a period of nine months. The two forensically important species examined were *Calliphora vicina* and *Lucilia sericata*. The hydrocarbons were chemically extracted and analysed using Gas Chromatography – Mass Spectrometry. Statistical analysis was then applied in the form of non-metric multidimensional scaling analysis (NMDS), permutational multivariate analysis of variance (PERMANOVA) and random forest models. This study was successful in determining age differences within the empty cases, which to date, has not been establish by any other technique.

## Introduction

Forensic entomology can be used to estimate the minimum Post Mortem Interval (PMI_min_) when insects associated with a body are examined, and the identity and age is determined. An experienced entomologist can establish the identification of Calliphoridae that were present using the empty puparial cases if they are in good condition but there are currently no means of ageing the empty cases. In scenarios where all other entomological evidence is no longer present, puparial cases can often be all that remains and therefore being able to establish the age could give an indication of the PMI^1–4^.

The presence of empty puparial cases is often associated with older corpses and can provide vital information in relation to PMI estimations. It can also give information of the presence of drugs that the larvae were exposed to, which can remain in the case once the adult has emerged^5^. At present, several papers have been published looking at using the larvae and pupae of forensically important blowflies to calculate the PMI estimations^4, 6–9^. However, few papers have looked into the possibility of using puparial cases and its potential has not yet been fully realised^10^. Although DNA can be used to identify empty puparial cases^5^, there are no such techniques that can age them. One research group from China analysed the cuticular hydrocarbons and the effects of weathering of *Chrysomya megacephala* to establish the age of the cases, but only preliminary observations were presented up to 90 days^1^. Another group in France compared the hydrocarbons and transesterified wax products of *Hydrotaea aenescens* over two time periods, 15 years apart^2^.

Cuticular hydrocarbons (CHC) are one of the components of the lipid wax layer of insect cuticles. CHCs are composed of long linear chains of hydrogen and carbon atoms with a chain length varying from C17 to C35. In insects, they are observed in their saturated and unsaturated form and can have one or more methyl groups attached to the chain length. In the saturated form, *n*-alkanes consist of all the carbons being joined together with single bonds but they may have one or more methyl groups present. In the unsaturated form, one or more double bonds maybe present along the length of the chain. They are believed to have different functions in different insects, but their main role is the prevention of desiccation^11^.

Previous studies on CHC on other insects have shown to be a successful identification^12–15^ and ageing tool^16–19^. Studies on forensically important blowflies have also proven to show great potential but these investigations are mainly presenting cuticular changes over time in relation to larvae^4, 20, 21^, with only two studies being published on trying to establish age related changes from the cuticular hydrocarbons^1, 2^. This is the first study to establish the age of empty puparial cases from two forensically important blowflies in Europe using CHCs analysed on Gas Chromatography-Mass Spectrometry (GC-MS), non-metric multidimensional scaling analysis (NMDS) and permutational multivariate analysis of variance (PERMANOVA).

This study presents preliminary results with the aim of ageing the empty puparial cases of two species of blowflies, *Calliphora vicina* and *Lucilia sericata* using their CHCs over a period of nine months.

## Results

### *L. sericata*: GC-MS analysis

The empty puparial cases were extracted weekly for the first 8 weeks then monthly until month 9.

In total, *L*. *sericata* yielded a profile of 24 identifiable compounds (with a peak area exceeding 0.5%) with some co-eluting for a total of 20 resolvable peaks from week 1 to month 9 (Table 6.5). The hydrocarbons (from week 1) consisted of *n*-alkanes (45%), alkenes (5%) and methyl branched hydrocarbons (50%). The chain lengths range from C25:H to C33:H.

**Figure 1:**
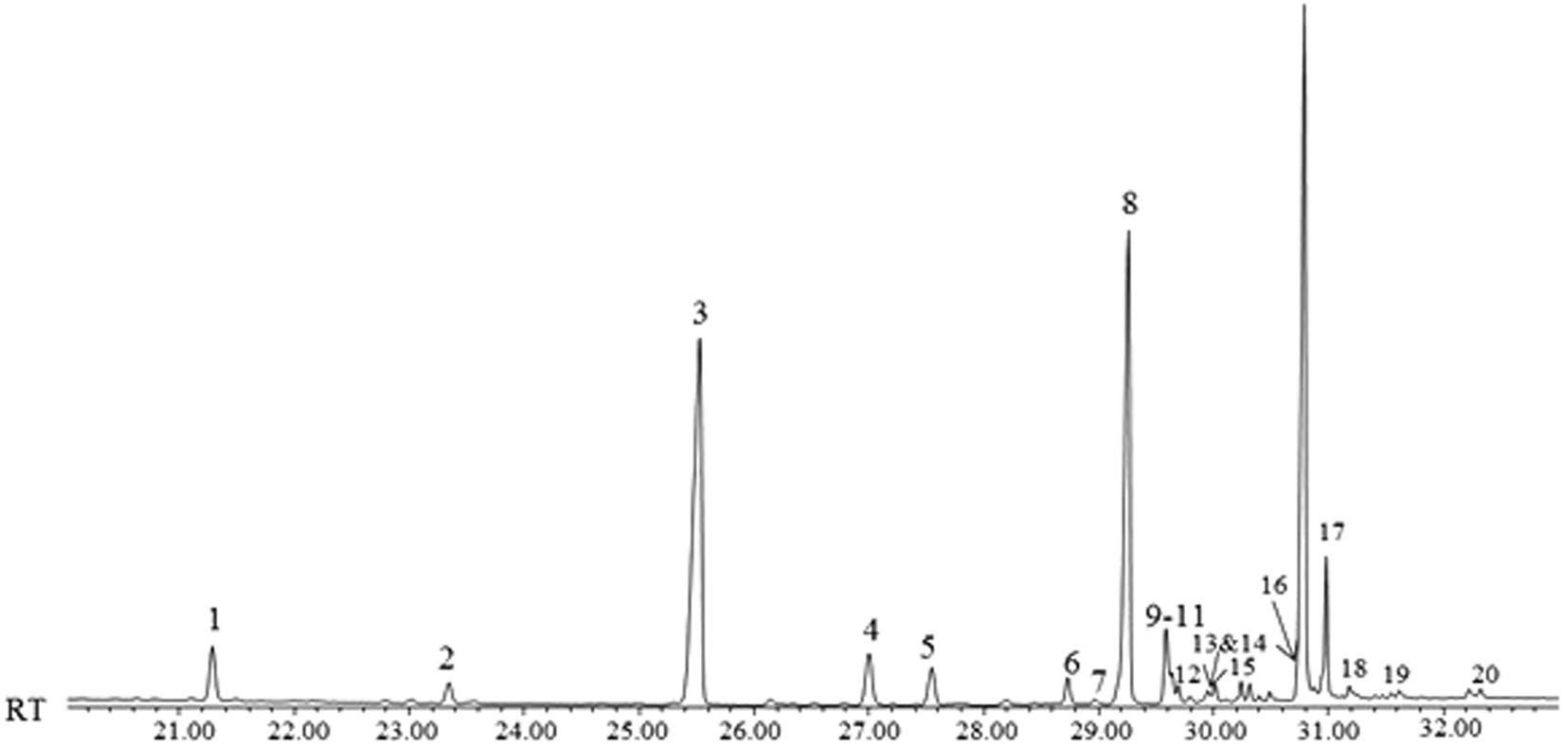
GC chromatogram of *L*. *sericata* puparial case extracted at week 1.

Figure 1 shows the GC chromatogram of a sample from Week 1. In comparison to the larvae profiles^22^, there are far fewer compounds present, and they are all high molecular weight hydrocarbons, resembling the profile of the adult fly.

In general, the odd *n*-alkanes exhibit much larger peak areas than the even *n*-alkanes, methyl branched and alkene compounds.

Table 1 lists the identified compounds extracted from the cuticle of the puparial case along with the total percentage of each compound present, the percentage standard deviation and the Kovats Indices.

**Table 1:** List of the compounds extracted from the puparial case and used for subsequent multivariate analysis of *L*. *sericata*, with the total percentage of each compound present, the percentage standard deviation for each week/month and the Kovats Indices to aid identification (peak numbers refer to Fig. 1).

**Peak number**	**Peak identification**	**Kovats iu**	**Week 1**	**Week 2**	**Week 3**	**Week 4**	**Week 5**	**Week 6**	**Week 7**	
			**n = 10%**	**n = 10%**	**n = 10%**	**n = 10%**	**n = 10%**	**n = 10**	**n = 10%**	
1	Pentacosane	2500	3.88 ± 1.00	8.14 ± 1.80	7.12 ± 1.19	7.48 ± 1.32	5.85 ± 4.54	5.60 ± 1.84	6.68 ± 2.34	
2	Hexacosane	2600	1.41 ± 0.36	1.25 ± 0.29	1.28 ± 0.34	1.36 ± 0.36	1.54 ± 0.23	1.92 ± 0.63	1.70 ± 0.43	
3	Heptacosane	2700	34.05 ± 7.75	34.33 ± 8.72	34.72 ± 8.08	34.48 ± 9.62	34.31 ± 5.40	47.82 ± 10.55	46.77 ± 11.26	
4	3-Methylheptacosane	2774	4.26 ± 1.08	5.08 ± 1.39	4.89 ± 1.44	4.44 ± 1.15	4.99 ± 1.10	7.36 ± 1.72	7.45 ± 1.93	
5	Octacosane	2800	2.96 ± 0.53	4.03 ± 1.07	4.19 ± 1.10	4.23 ± 1.21	4.55 ± 0.68	2.70 ± 1.00	2.55 ± 0.75	
6	2-Methyloctacosane	2869	1.62 ± 0.59	2.51 ± 0.96	2.17 ± 0.56	2.63 ± 1.02	2.48 ± 0.52	2.35 ± 0.77	2.38 ± 0.66	
7	Nonacosene^2^	2883	0.90 ± 0.42	tr	tr	tr	tr	tr	tr	
8	Nonacosane	2900	31.99 ± 3.68	30.19 ± 6.98	32.48 ± 8.04	32.78 ± 7.24	32.99 ± 5.88	18.63 ± 5.23	17.92 ± 4.86	
9	11 + 15-Methylnonacosane	2935	3.23 ± 0.92	4.66 ± 2.34	2.80 ± 0.55	3.59 ± 1.12	2.76 ± 1.20	4.56 ± 1.49	4.52 ± 2.28	
10	9-Methylnonacosane	2940	1.12 ± 0.30	0.97 ± 0.71	0.34 ± 0.29	0.33 ± 0.28	0.55 ± 0.55	1.76 ± 0.60	2.04 ± 0.77	
11	7-Methylnonacosane	2946	0.71 ± 0.19	1.53 ± 0.61	1.57 ± 0.40	1.54 ± 0.46	1.55 ± 0.33	1.65 ± 0.55	1.85 ± 0.61	
12	5-Methylnonacosane	2955	tr	0.70 ± 0.27	0.75 ± 0.25	0.70 ± 0.24	0.83 ± 0.18	0.48 ± 0.24	0.63 ± 0.17	
13	9,17-Dimethylnonacosane^1^	2971	0.64 ± 0.14	0.13 ± 0.06	1.43 ± 0.41	0.15 ± 0.15	0.72 ± 0.13	1.11 ± 0.46	1.35 ± 0.41	
14	3-Methylnonacosane	2977	0.83 ± 0.19	1.39 ± 0.43	1.39 ± 0.41	1.13 ± 0.49	1.43 ± 0.24	1.29 ± 0.46	1.49 ± 0.42	
15	Triacontane	3000	1.26 ± 0.19	0.65 ± 0.15	0.63 ± 0.21	0.67 ± 0.19	0.93 ± 0.22	0.26 ± 0.14	0.26 ± 0.07	
16	2-Methyltriacontane	3067	tr	1.13 ± 0.47	1.00 ± 0.30	1.15 ± 0.49	1.03 ± 0.17	0.81 ± 0.34	0.97 ± 0.27	
17	Hentriacontane	3100	8.32 ± 1.65	2.34 ± 0.75	2.14 ± 1.00	2.15 ± 0.79	2.25 ± 0.74	0.90 ± 0.34	0.57 ± 0.47	
18	15 + 13 + 11 + 9-Methylhentriacontane	3132	0.95 ± 0.31	0.40 ± 0.30	0.35 ± 0.18	0.41 ± 0.21	0.43 ± 0.15	0.35 ± 0.14	0.47 ± 0.17	
19	Dotriacontane	3200	1.00 ± 0.17	0.24 ± 0.03	0.40 ± 0.14	0.37 ± 0.19	0.41 ± 0.18	0.31 ± 0.06	0.25 ± 0.04	
20	Tritriacontane	3300	0.88 ± 0.21	0.34 ± 0.05	0.36 ± 0.09	0.40 ± 0.09	0.39 ± 0.09	0.16 ± 0.05	0.15 ± 0.06	
**Peak number**	**Peak identification**	**Kovats iu**	**Week 8**	**Month 3**	**Month 4**	**Month 5**	**Month 6**	**Month 7**	**Month 8**	**Month 9**
			**n = 10%**	**n = 10%**	**n = 10%**	**n = 10%**	**n = 10%**	**n = 10%**	**n = 10%**	**n = 10%**
1	Pentacosane	2500	5.41 ± 1.03	1.02 ± 0.36	0.63 ± 0.29	0.67 ± 0.26	0.97 ± 0.33	2.61 ± 1.06	1.25 ± 0.37	0.94 ± 0.69
2	Hexacosane	2600	1.73 ± 0.40	1.61 ± 0.50	1.12 ± 0.49	1.42 ± 0.19	1.33 ± 0.31	1.38 ± 0.20	1.47 ± 0.55	1.30 ± 0.70
3	Heptacosane	2700	49.51 ± 11.44	43.34 ± 14.03	34.15 ± 17.00	39.16 ± 6.63	37.27 ± 7.66	33.08 ± 6.69	34.81 ± 12.70	33.79 ± 18.92
4	3-Methylheptacosane	2774	7.24 ± 1.14	4.57 ± 1.71	4.31 ± 1.49	4.20 ± 0.65	4.05 ± 1.25	3.80 ± 0.57	3.80 ± 1.22	4.12 ± 2.65
5	Octacosane	2800	2.10 ± 1.25	4.39 ± 1.60	4.56 ± 1.39	4.20 ± 0.76	4.10 ± 0.77	3.49 ± 0.75	3.67 ± 1.40	3.91 ± 2.25
6	2-Methyloctacosane	2869	2.31 ± 0.50	2.23 ± 0.93	2.72 ± 0.91	2.60 ± 0.58	2.07 ± 0.31	2.07 ± 0.33	2.26 ± 0.83	2.27 ± 1.08
7	Nonacosene^2^	2883	tr	tr	tr	tr	tr	tr	tr	tr
8	Nonacosane	2900	18.18 ± 4.03	30.39 ± 11.24	34.46 ± 11.13	30.73 ± 6.17	33.35 ± 7.05	30.55 ± 6.94	32.60 ± 12.19	32.38 ± 16.50
9	11 + 15-Methylnonacosane	2935	4.31 ± 0.90	3.93 ± 1.62	5.47 ± 2.64	4.89 ± 0.85	4.08 ± 1.78	5.19 ± 1.42	4.21 ± 1.10	4.63 ± 1.46
10	9-Methylnonacosane	2940	1.72 ± 0.27	1.45 ± 0.58	1.40 ± 0.48	1.39 ± 0.23	1.65 ± 0.84	2.03 ± 0.46	1.65 ± 0.47	1.63 ± 0.65
11	7-Methylnonacosane	2946	1.62 ± 0.29	1.26 ± 0.47	1.34 ± 0.53	1.27 ± 0.19	1.35 ± 0.93	1.76 ± 0.33	1.47 ± 0.41	1.49 ± 0.62
12	5-Methylnonacosane	2955	0.52 ± 0.13	0.54 ± 0.29	0.74 ± 0.24	0.63 ± 0.08	0.54 ± 0.24	0.75 ± 0.12	0.72 ± 0.18	0.77 ± 0.44
13	9,17-Dimethylnonacosane^1^	2971	1.15 ± 0.27	0.53 ± 0.25	0.31 ± 0.11	0.24 ± 0.10	0.21 ± 0.12	0.66 ± 0.15	0.63 ± 0.17	0.70 ± 0.34
14	3-Methylnonacosane	2977	1.37 ± 0.22	1.25 ± 0.50	1.56 ± 0.63	1.53 ± 0.24	1.70 ± 0.96	1.26 ± 0.23	1.40 ± 0.44	1.51 ± 0.93
15	Triacontane	3000	0.23 ± 0.09	0.49 ± 0.25	0.83 ± 0.31	0.69 ± 0.15	1.14 ± 1.08	1.00 ± 0.28	0.89 ± 0.32	1.03 ± 0.45
16	2-Methyltriacontane	3067	0.91 ± 0.22	1.09 ± 0.61	1.76 ± 0.96	1.96 ± 0.57	1.53 ± 0.53	1.05 ± 0.26	1.31 ± 0.47	1.45 ± 0.81
17	Hentriacontane	3100	0.80 ± 0.36	1.04 ± 0.51	2.96 ± 0.86	2.72 ± 0.95	3.06 ± 0.64	5.27 ± 1.84	4.27 ± 1.26	4.79 ± 1.39
18	15 + 13 + 11 + 9-Methylhentriacontane	3132	0.47 ± 0.09	0.25 ± 0.16	0.81 ± 0.35	0.78 ± 0.20	0.65 ± 0.24	1.64 ± 0.74	1.53 ± 0.38	1.12 ± 0.45
19	Dotricontane	3200	0.25 ± 0.06	0.39 ± 0.12	0.31 ± 0.18	0.31 ± 0.09	0.28 ± 0.08	1.13 ± 0.40	0.95 ± 0.37	0.95 ± 0.41
20	Tritriacontane	3300	0.16 ± 0.04	0.21 ± 0.06	0.55 ± 0.31	0.61 ± 0.25	0.68 ± 0.06	1.27 ± 0.24	1.10 ± 0.44	1.21 ± 0.42

^1^Tentative identification based on Kovats Index. ^2^Double bond position determined but not assigned to specific peaks.

### *L. sericata*: Multivariate analysis

**Figure 2:**
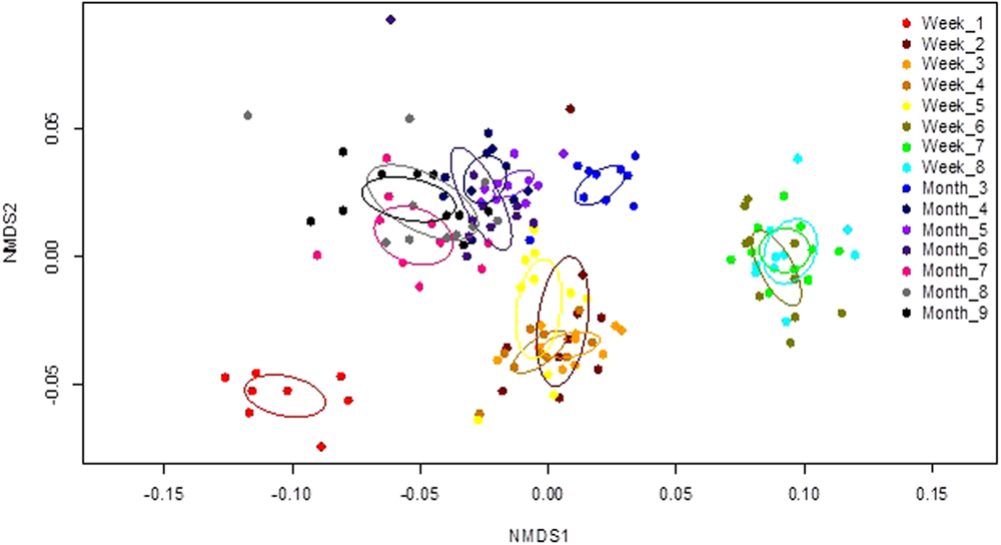
*Lucilia sericata* hydrocarbon profiles of puparial cases aged from 1 week to 9 months were visualized using a non-metric multidimensional scaling ordination based on a Bray-Curtis dissimilarity matrix and plotted on two dimensions (stress = 0.138, R^2^ = 0.926); each puparial case age had a 95% confidence ellipses around class centroids.

**Figure 3:**
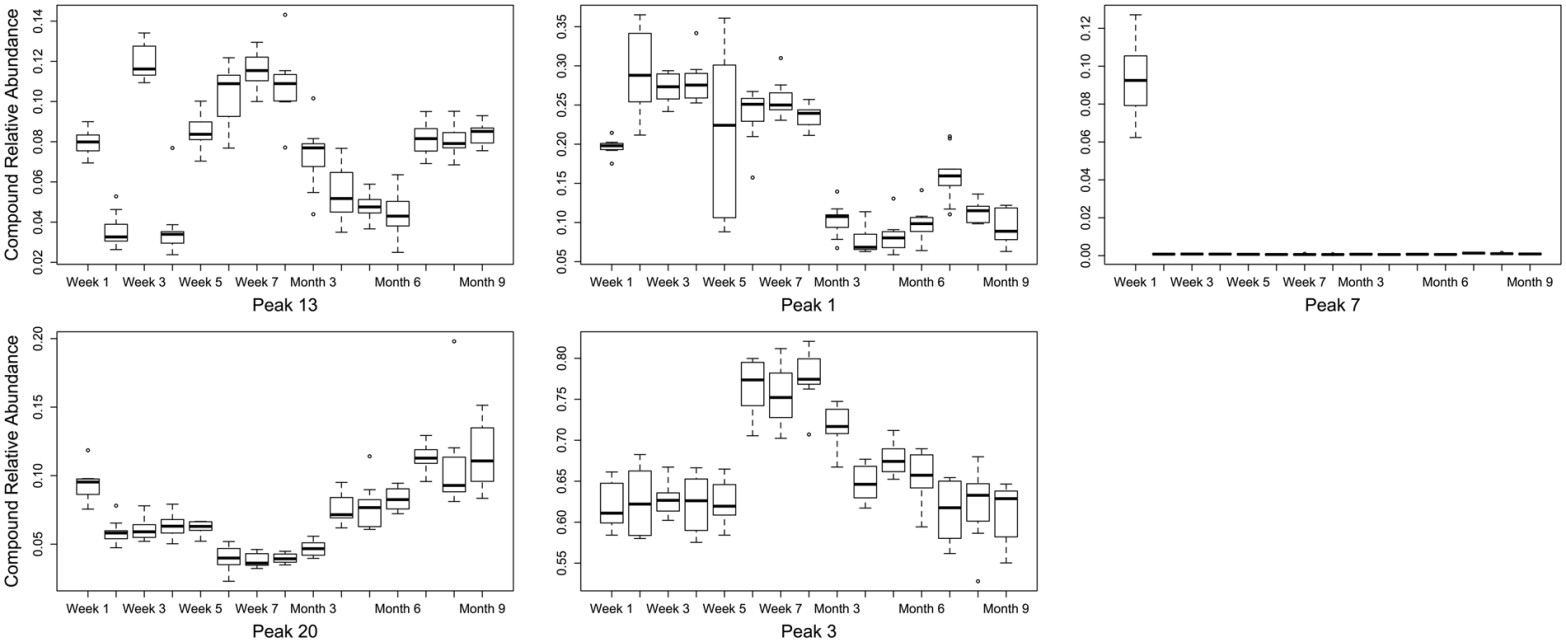
The top five hydrocarbon compounds identified that contributed to the most variation in *Lucilia sericata* puparial case age (1 week to 9 months): 9,17-diMethylnonacosane (Peak 13), Pentacosane (Peak 1), Nonacosene (Peak 7), Tritriacontane (Peak 20), and Heptacosane (Peak 3). The solid black line across the color box represents the median value, while a hollow circle indicates an outlier value.

There were significant cuticular hydrocarbon profile differences (P < 0.001) among puparial case ages for each species (Table 2). A two-dimensional NMDS ordination explained 92.6% (stress = 0.138) of hydrocarbon structure for aged *L*. *sericata* puparial cases (Fig. 2), with distinct clusters based on cohorts of similar age for week 1, weeks 2–5, weeks 6–8, month 3, months 4–6, and months 7–9. Further, five hydrocarbon compounds were identified to be important for discriminating among the discrete age groups during the *L*. *sericata* puparial case aging process (Fig. 3): 9,17-diMethylnonacosane (Peak 13) demonstrated a modified sine curve trend with the highest relative abundance during weeks 3 and 7; Pentacosane (Peak 1) levels were elevated until week 8 with a decline in relative abundance from month 3–9; Nonacosene (Peak 7) was the most abundant during the first week of aging; Tritriacontane (Peak 20) demonstrated a u-shaped curve with the most elevated relative abundance levels occurring during months 7–9; and Heptacosane (Peak 3) levels peaked during weeks 6–8 with a decline in relative abundance during the remain process until month 9.

**Table 2:** PERMANOVA results testing *Lucilia sericata* and *Calliphora vicina* hydrocarbon profiles based on Bray-Curtis dissimilarity among aged puparial cases from 1–8 weeks and 3–9 months.

Source	d.f.	*SS*	MS	F	R^2^	P
*Lucilia sericata*	14	0.767	0.055	35.001	0.787	0.001
Residuals	133	0.209	0.002		0.214	
Total	147	0.975			1.000	
*Calliphora vicina*	14	0.402	0.0287	33.942	0.780	0.001
Residuals	134	0.113	0.001		0.210	
Total	148	0.515			1.000

Although it is very difficult to visualise any changes occurring within the GC chromatograms over time, small changes were present and multivariate analysis allowed for these slight variations to be visualised. Age-related changes over time were observed for the puparial cases of *L*. *sericata*. Therefore the results show some potential for hydrocarbon analysis to be used in PMI estimations of older cadavers, when other entomology evidence may not be present.

A useful week 1 age indicator is C29:1 (compound 7), which is specific to this age, and is likely to explain why this week is the only week to group individually in Fig. 2. The percentage for C27:H and C29:H are relatively stable with the exception of weeks 6 to 8 and month 3, where there is more variation. Week 6 to 8 are seen to group together within plot shown in Fig. 2.

Results were presented up to month 9 and four age groups were established using NMDS, consisting of week 1, weeks 2 to 5, weeks 6 to 8 and finally a much larger group containing months 3 and then months 4 to 9 (moving across the NMDS1 axis from right to left).

### *C. vicina*: GC-MS Analysis

The hydrocarbon profile of *C*. *vicina* puparial cases contained 37 compounds with some co-eluting giving a total of 31 resolvable peak from week 1 to month 9 (Table 3). The hydrocarbons consisted of *n*-alkanes (29%), alkenes (3%) and methyl branched alkanes (68%) in the form of mono-, di- and tri-methyl alkanes. The chain lengths ranged from C25:H to C33:H.

**Table 3:** List of all compounds extracted from the puparial case of *C*. *vicina* and the Kovats Index to aid identification.

Peak number	Peak Identification	Kovats iu
1	Pentacosane	2500
2	**3-Methylpentacosane**	2574
3	Hexacosane	2600
4	**2-Methylhexacosane**	2661
5	Heptacosane	2700
6	**11 + 13-Methylheptacosane**	2731
7	**9-Methylheptacosane**	2734
8	**7-Methylheptacosane**	2740
9	**5-Methylheptacosane**	2749
10	**11,15-Dimethylheptacosane** ^**1**^	2762
11	**3-Methylheptacosane**	2775
12	*5,x-Dimethylheptacosane ^**x**^	2783
13	Octacosane	2800
14	**Trimethylpentacosane** ^**2**^	2814
15	**12 + 15 + 16-Methyloctacosane**	2839
16	**8-Methyloctacosane**	2846
17	**2-Methyloctacosane**	2869
18	**x,10/x,12/x,14-Dimethyloctacosane** ^**y**^	2881
19	Nonacosane	2900
20	**4,8,12-Trimethyloctacosane** ^**1**^	2922
21	**11 + 13-Methylnonacosane**	2937
22	**7-Methylnonacosane**	2946
23	**5-Methylnonacosane**	2955
24	**11,15-Dimethylnonacosane** ^**1**^	2964
25	**9,15-Dimethylnonacosane** ^**1**^	2971
26	**3-Methylnonacosane**	2977
27	Triacontane	3000
28	**Hentriacontene** ^**3**^	3085
29	Hentriacontane	3100
30	Dotriacontane	3200
31	Tritriacontane	3300

Compounds in bold were used for subsequent multivariate analysis.

^1^Tentative identification based on Kovats Index.

^2^Methyl branch position not determined.

^3^Double bond positions only determined in adult flies.

^x^x = 9,11, 15.

^y^x = methyl branched position not determined.

**Figure 4:**
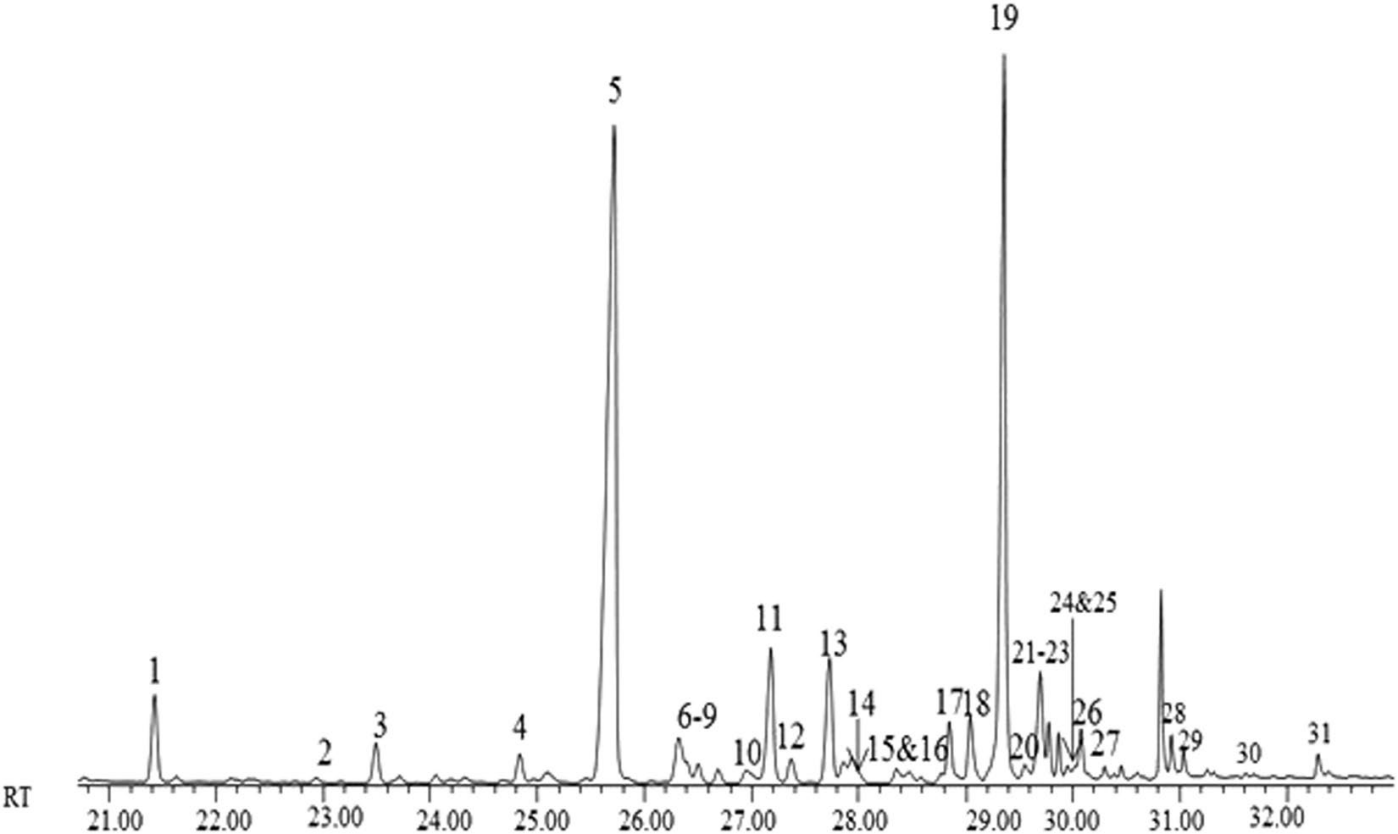
GC chromatogram of *C*. *vicina* puparial case extracted at week 1.

Figure 4 shows the chemical profile extracted from a week 1 puparial case of *C*. *vicina* with all peaks numbered and identified (Table 3).

There is considerable fluctuation within the peak areas over time across the compounds used for NMDS (Table 4 – *n*-alkanes removed). A group of compounds exhibiting substantially higher peak areas over others are 11 + 13-Methylheptacosane (peak 6), 3-Methylheptacosane (peak 11) and 11 + 13-Methylnonacosane (peak 21).

**Table 4:** List of the compounds extracted and used for subsequent multivariate analysis from the puparial cases of *C*. *vicina*, with the total percentage of each compound present, the percentage standard deviation for each week/month and the Kovats Indies to aid identification.

Peak number	Peak Identification	Kovats iu	Week 1	Week 2	Week 3	Week 4	Week 5	Week 6	Week 7	Week 8
			n = 10%	n = 10%	n = 10%	n = 10%	n = 10%	n = 10%	n = 10%	n = 10%
2	3-Methylpentacosane	2574	0.68 ± 0.28	3.19 ± 1.10	3.01 ± 1.13	3.07 ± 1.10	2.82 ± 1.54	3.07 ± 1.14	3.05 ± 0.78	2.91 ± 0.53
4	2-Methylhexacosane	2661	2.65 ± 1.73	1.60 ± 0.38	2.34 ± 0.62	1.97 ± 0.24	1.97 ± 0.60	2.52 ± 0.87	2.57 ± 1.03	2.38 ± 0.54
6	11 + 13-Methylheptacosane	2731	6.53 ± 2.69	7.53 ± 1.75	6.97 ± 1.71	7.97 ± 1.94	7.11 ± 2.04	11.57 ± 4.64	9.65 ± 2.18	10.44 ± 2.47
7	9-Methylheptacosane	2734	2.08 ± 0.70	1.77 ± 0.47	1.86 ± 0.53	1.87 ± 0.60	1.93 ± 0.57	2.35 ± 1.31	2.15 ± 0.46	2.28 ± 0.74
8	7-Methylheptacosane	2740	2.54 ± 0.86	3.43 ± 0.74	3.23 ± 0.80	3.25 ± 0.87	3.36 ± 1.09	4.36 ± 1.77	4.02 ± 0.89	4.04 ± 0.73
9	5-Methylheptacosane	2749	2.14 ± 0.79	2.92 ± 0.78	2.88 ± 0.66	2.84 ± 0.53	2.83 ± 1.05	3.06 ± 1.03	2.96 ± 0.59	3.07 ± 0.58
10	11,15-Dimethylheptacosane^1^	2762	2.19 ± 0.57	2.33 ± 0.54	2.40 ± 0.50	2.58 ± 0.58	2.27 ± 1.00	3.05 ± 0.55	2.90 ± 0.53	2.95 ± 0.73
11	3-Methylheptacosane	2775	15.39 ± 2.47	27.58 ± 8.66	27.55 ± 6.60	27.38 ± 6.90	27.27 ± 11.50	21.78 ± 3.82	24.51 ± 5.05	23.29 ± 4.87
12	*5,x-Dimethylheptacosane^1^	2783	3.80 ± 1.21	2.78 ± 0.71	2.71 ± 0.74	2.72 ± 0.70	2.68 ± 0.92	3.49 ± 0.87	3.22 ± 0.70	3.47 ± 0.86
14	Trimethylpentacosane^2^	2814	3.62 ± 1.15	3.53 ± 0.84	3.79 ± 0.90	2.99 ± 1.04	4.60 ± 2.80	1.49 ± 0.27	1.27 ± 0.27	1.32 ± 0.30
15	12 + 15 + 16-Methyloctacosane	2839	2.68 ± 1.25	1.81 ± 0.42	1.89 ± 0.43	2.06 ± 0.41	2.12 ± 0.76	2.46 ± 0.68	2.13 ± 0.45	2.40 ± 0.63
16	8-Methyloctacosane	2846	2.34 ± 0.87	2.62 ± 0.75	2.62 ± 0.70	2.58 ± 0.89	2.40 ± 1.15	3.31 ± 0.77	3.24 ± 0.71	3.24 ± 0.60
17	2-Methyloctacosane	2869	7.45 ± 2.88	0.85 ± 0.28	0.83 ± 0.27	0.91 ± 0.27	1.07 ± 0.60	0.65 ± 0.11	0.76 ± 0.21	0.71 ± 0.21
18	x,10/x,12/x,14-Dimethyloctacosane^1^	2881	9.36 ± 2.26	3.00 ± 0.68	3.17 ± 0.68	3.01 ± 0.64	2.72 ± 1.10	5.49 ± 0.83	5.87 ± 1.49	5.22 ± 1.69
20	4,8,12-Trimethyloctacosane^1^	2922	3.02 ± 1.20	2.88 ± 0.91	2.74 ± 0.76	2.83 ± 1.10	2.58 ± 1.39	3.77 ± 0.89	3.85 ± 0.92	3.85 ± 0.69
21	11 + 13-Methylnonacosane	2937	15.96 ± 5.09	12.29 ± 3.29	12.79 ± 2.84	12.35 ± 3.42	11.87 ± 4.58	14.67 ± 3.04	14.39 ± 2.84	14.88 ± 2.96
22	7-Methylnonacosane	2946	3.83 ± 0.55	3.04 ± 0.85	3.10 ± 0.64	3.04 ± 0.63	3.15 ± 0.99	3.07 ± 0.73	3.28 ± 0.57	3.35 ± 0.58
23	5-Methylnonacosane	2955	2.60 ± 0.22	1.77 ± 0.52	1.85 ± 0.46	2.01 ± 0.55	1.90 ± 0.73	1.41 ± 0.30	1.63 ± 0.38	1.60 ± 0.40
24	11,15-Dimethylnonacosane^1^	2964	1.55 ± 0.46	1.51 ± 0.58	1.37 ± 0.43	1.41 ± 0.61	1.23 ± 0.66	1.41 ± 0.29	1.56 ± 0.34	1.47 ± 0.26
25	9,15-Dimethylnonacosane^1^	2971	1.23 ± 0.40	1.47 ± 0.34	1.54 ± 0.37	1.60 ± 0.54	1.44 ± 0.56	2.11 ± 0.34	1.98 ± 0.40	2.05 ± 0.58
26	3-Methylnonacosane	2977	4.57 ± 0.85	3.83 ± 1.12	3.90 ± 0.82	3.97 ± 0.92	3.84 ± 1.35	3.70 ± 0.69	3.84 ± 0.66	3.89 ± 0.80
28	Hentriacontene^3^	3085	3.78 ± 0.83	8.30 ± 3.28	7.47 ± 2.11	7.60 ± 2.45	8.85 ± 2.06	1.22 ± 0.15	1.18 ± 0.40	1.19 ± 0.24
**Peak number**	**Peak Identification**	**Kovats iu**	**Month 3**	**Month 4**	**Month 5**	**Month 6**	**Month 7**	**Month 8**	**Month 9**	
			**n = 10%**	**n = 10%**	**n = 10%**	**n = 10%**	**n = 10%**	**n = 10%**	**n = 10%**	
2	3-Methylpentacosane	2574	2.75 ± 0.59	3.13 ± 0.72	2.87 ± 1.14	2.03 ± 0.55	2.16 ± 0.63	2.20 ± 0.23	1.94 ± 0.46	
4	2-Methylhexacosane	2661	3.81 ± 10.65	0.39 ± 0.12	2.46 ± 0.95	1.78 ± 0.53	1.81 ± 0.37	1.94 ± 0.45	1.86 ± 0.32	
6	11 + 13-Methylheptacosane	2731	10.20 ± 2.05	10.15 ± 1.86	10.44 ± 4.10	7.59 ± 2.45	7.96 ± 2.15	8.59 ± 1.85	9.01 ± 2.05	
7	9-Methylheptacosane	2734	2.32 ± 0.64	2.28 ± 0.48	2.40 ± 0.95	1.55 ± 0.59	1.66 ± 0.50	1.78 ± 0.34	2.06 ± 0.35	
8	7-Methylheptacosane	2740	3.93 ± 0.69	4.03 ± 0.78	4.12 ± 1.60	3.04 ± 1.01	3.17 ± 0.85	3.33 ± 0.65	3.59 ± 0.74	
9	5-Methylheptacosane	2749	2.75 ± 0.52	3.05 ± 0.58	3.02 ± 1.16	2.80 ± 1.56	2.54 ± 0.68	2.54 ± 0.40	2.43 ± 0.55	
10	11,15-Dimethylheptacosane^1^	2762	3.00 ± 0.63	3.03 ± 0.60	2.98 ± 1.17	2.44 ± 0.77	2.53 ± 0.63	2.59 ± 0.51	2.35 ± 0.47	
11	3-Methylheptacosane	2775	22.80 ± 4.06	23.68 ± 4.45	23.26 ± 8.94	19.43 ± 5.57	21.13 ± 6.07	20.20 ± 2.46	18.71 ± 4.53	
12	5,x-Dimethylheptacosane^x^	2783	3.50 ± 1.04	3.44 ± 0.79	3.53 ± 1.38	2.76 ± 0.90	2.66 ± 0.63	2.84 ± 0.68	3.14 ± 0.57	
14	Trimethylpentacosane^2^	2814	1.09 ± 0.20	1.13 ± 0.24	1.55 ± 0.67	3.54 ± 1.09	3.33 ± 0.75	3.42 ± 0.85	4.07 ± 0.99	
15	12 + 15 + 16-Methyloctacosane	2839	3.81 ± 0.75	3.87 ± 0.90	4.16 ± 1.54	1.93 ± 0.63	1.97 ± 0.49	2.07 ± 0.47	2.11 ± 0.42	
16	8-Methyloctacosane	2846	3.07 ± 0.57	3.01 ± 0.70	3.02 ± 1.20	3.15 ± 1.55	2.61 ± 0.69	2.69 ± 0.63	2.96 ± 0.62	
17	2-Methyloctacosane	2869	0.65 ± 0.17	0.70 ± 0.14	0.66 ± 0.29	7.05 ± 2.80	6.22 ± 1.43	6.82 ± 1.85	6.60 ± 1.29	
18	x,10/x,12/x,14-Dimethyloctacosane^y^	2881	5.42 ± 0.92	6.06 ± 1.41	4.81 ± 1.90	6.59 ± 1.77	6.80 ± 1.81	5.78 ± 1.13	4.36 ± 1.30	
20	4,8,12-Trimethyloctacosane^1^	2922	3.65 ± 0.78	3.58 ± 0.88	3.59 ± 1.44	4.26 ± 1.32	3.62 ± 0.94	3.54 ± 0.90	3.91 ± 0.91	
21	11 + 13-Methylnonacosane	2937	14.15 ± 2.25	14.58 ± 2.62	14.34 ± 5.55	14.04 ± 4.02	13.95 ± 3.61	14.62 ± 3.31	16.48 ± 3.33	
22	7-Methylnonacosane	2946	3.11 ± 0.52	3.40 ± 0.58	3.27 ± 1.25	3.18 ± 1.01	3.25 ± 0.95	3.26 ± 0.60	3.66 ± 0.83	
23	5-Methylnonacosane	2955	1.62 ± 0.49	1.67 ± 0.28	1.63 ± 0.65	1.84 ± 0.62	1.92 ± 0.62	1.73 ± 0.26	1.64 ± 0.46	
24	11,15-Dimethylnonacosane^1^	2964	1.60 ± 0.50	1.43 ± 0.25	1.31 ± 0.55	1.69 ± 0.56	1.50 ± 0.41	1.51 ± 0.33	1.58 ± 0.40	
25	9,15-Dimethylnonacosane^1^	2971	1.94 ± 0.36	2.13 ± 0.47	2.10 ± 0.79	2.46 ± 0.93	2.17 ± 0.73	2.27 ± 0.49	2.30 ± 0.53	
26	3-Methylnonacosane	2977	3.84 ± 0.65	3.97 ± 0.68	3.94 ± 1.51	4.22 ± 1.28	4.14 ± 1.10	4.14 ± 0.70	4.38 ± 1.01	
28	Hentriacontene^3^	3085	1.01 ± 0.23	1.29 ± 0.33	0.53 ± 0.26	2.63 ± 0.77	2.89 ± 0.89	2.17 ± 0.53	0.87 ± 0.36	

^1^Tentative identification based on Kovats Index.

^2^Methyl branch position not determined.

^3^Double bond positions only determined for adult flies.

^x^x = 9, 11, 15.

^y^x = methyl branched position not determined.

C31:1 (compound 28) is the only alkene present in the profile and is detected at its most abundant in week 5 before the concentration drops for the remainder of the extraction period.

When looking at the chromatograms of the varying ages across the extraction period, very few distinctions can be made and ageing from observing the hydrocarbon profiles alone is not possible.

### *C. vicina*: Multivariate analysis

Of the 31 resolvable peaks extracted from the cuticle of the puparial cases, 22 were used for multivariate analysis. Enhanced NMDS results were obtained when the *n*-alkanes were excluded from the dataset. The methyl branched compounds consisted of 66% and the alkenes only contributed 3% to the total number of hydrocarbons present. The *n*-alkanes were removed to reduce the scatter and enhance the results.

**Figure 5:**
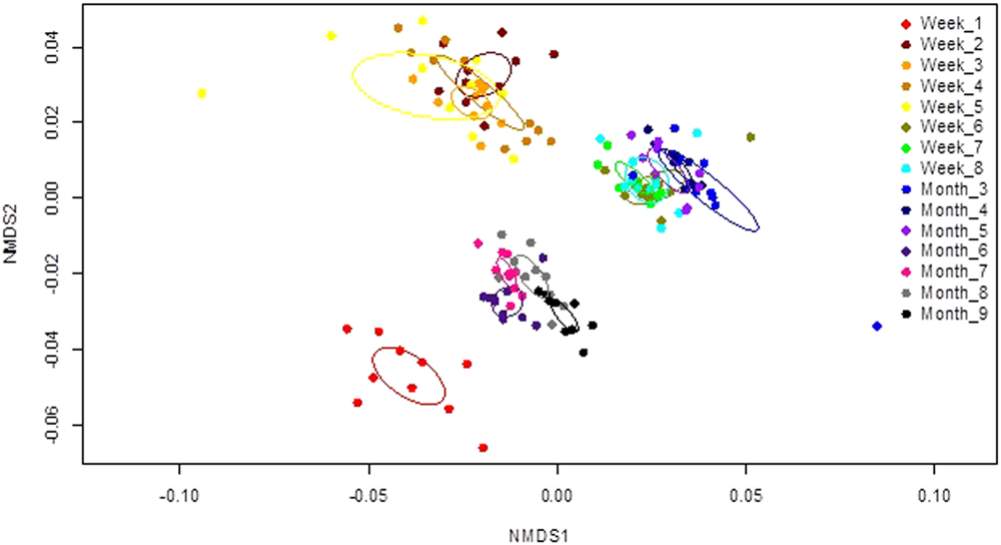
*Calliphora vicina* hydrocarbon profiles of puparial cases aged from 1 week to 9 months were visualized using a non-metric multidimensional scaling ordination based on a Bray-Curtis dissimilarity matrix and plotted on two dimensions (stress = 0.138, R^2^ = 0.967); each puparial case age had a 95% confidence ellipses around class centroids.

**Figure 6:**
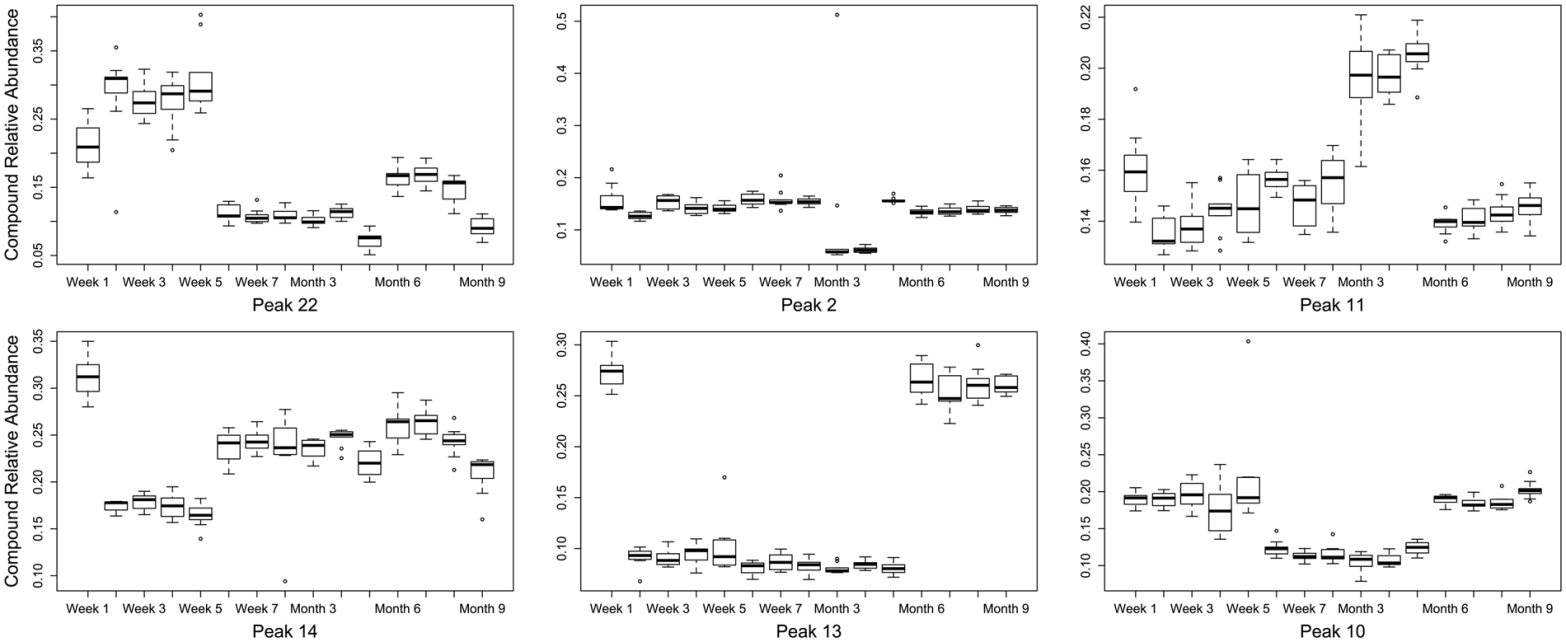
The top six hydrocarbon compounds identified that contributed to the most variation in *Calliphora vicina* puparial case age (1 week to 9 months): Hentriacontene (Peak 22), 2-Methylhexacosane (Peak 2), 12 + 15 + 16-Methyloctacosane (Peak 11), x,10/x,12/x,14di-Methyloctacosane (Peak 14), 2-Methyloctacsane (Peak 13), and Trimethylpentacosane (Peak 10). The solid black line across the color box represents the median value, while a hollow circle indicates an outlier value.

*C*. *vicina* aged puparial cases demonstrated similar results to *L*. *sericata*; a two-dimensional NMDS ordination explained 96.7% (stress = 0.138) of hydrocarbon structure for aged puparial cases (Fig. 5). Four distinct clusters based on cohorts of similar aged *C*. *vicina* puparial cases were identified based on hydrocarbon structure: week 1, weeks 2–5, weeks 6–8 and month 3, and months 4–9. Additionally, six hydrocarbon compounds were identified as important for discriminating among the discrete age groups during the *C*. *vicina* puparial case aging (Fig. 6), which were different from those identified in *L*. *sericata*. There was an elevated relative abundance of Hentriacontene (Peak 22) during the first 5 weeks of puparial case aging, while 2-Methylhexacosane (Peak 2) demonstrated a decrease in relative abundance during months 3 and 4. Also, 12 + 15 + 16-Methyloctacosane (Peak 11), x,10/x,12/x,14di-Methyloctacosane (Peak 14), and 2-Methyloctacsane (Peak 13) had elevated relative abundance levels during the first week of aging. After the first week, the three aforementioned hydrocarbon compounds displayed various relative abundance patterns: 12 + 15 + 16-Methyloctacosane increased until month 5 with peak levels occurring in months 3–5 followed by a decrease in the remaining three months; x,10/x,12/x,14di-Methyloctacosane followed a pattern of increasing relative abundance until month 7 followed by a decline until month 9; and 2-Methyloctacsane had consistently decreased levels until month 6–9, which had an elevation in 2-Methyloctacsane that approximately matched those levels detected in week 1. Finally, Trimethylpentacosane (Peak 10) demonstrated a u-shaped trend with relative abundance amounts remaining consistent during weeks 1–5 and months 6–9 with a decrease in relative abundance from week 6 to month 5.

Four groups are apparent from the NMDS plot in Fig. 5. These groups contain extracts from week 1, which clusters individually to the other extracts as one group. Weeks 2 to 5 cluster together as does week 6 to month 5 and finally months 6 to 9.

## Discussion

Puparial cases are often overlooked at crime scenes because little information about their age can be established. However, in scenes where puparial cases are the only entomological evidence present, being able to age them or have an indication of their age would be extremely advantageous. The results presented here for *L*. *sericata* and *C*. *vicina* puparial cases show the great potential to use hydrocarbons to age a life stage that presently cannot be aged using any other technique. Although there are very few published papers on empty puparial cases analysis, there has been work carried out to investigate the age of forensically important pupae. It is extremely difficult to age pupae using morphological changes, hence DNA techniques are being developed to look into ageing this life stage using gene expression^6, 23^ with a good degree of success. Davies & Harvey^24^ successfully aged *C*. *vicina* pupae using internal morphological analysis and there is some very promising work published by Richards and co-workers^25^ using Micro-computed tomography (micro-CT) to image developing flies based on a combination of the external and internal morphological markers. This technique enabled pupae to be imaged multiple times during their development with no adverse effects.

The above literaure highlights some techniques that are successfully being utilised to age the pupae but not for the empty puparial cases, where a literature search highlights there is currently no other means to age them, other than cuticular hydrocarbon analysis^1, 2^, emphasising the importance of this study.

To our knowledge the only other studies investigating the changes in the cuticular hydrocarbon profile of puparial cases, is a paper published by Zhu and co-workers^1^ and more recent work by Frere *et al*.^2^. Zhu *et al*. presented results from the puparial cases of *Chrysomya megacephala*, up to 90 days, to test the effect that weathering may have on the hydrocarbons. Similar to these results, they were able to determine a number of significant time-dependant changes within the hydrocarbon profile, therefore highlighting the potential hydrocarbons have to be used to age the empty puparial cases and subsequently aiding PMI estimations^1^. Frere *et al*. had a slightly different approach by examining recent (2012) and old puparia (1997) by analysing the puparia hydrocarbons and transesterified wax products, and comparing the two sets of results from the same fly species, *Hydrotaea aenescens*. Their results showed some differences within the hydrocarbon and fatty acid esters in comparison to the transesterified waxes, with similar trends observed to those reported by Zhu *et al*., but with the examined sample sets being 15 years apart, there was no ageing followed over a shorter period of time, which is more realistic to what you might find at a scene.

In this study the time period that we looked at was far longer (up to 9 months) than investigsted by Zhu and co-workers^1^, and it was clear that observations of the GC chromatograms alone cannot be used to determine the ages of the cases; there are very few significant trends when looking at the peak area percentages of the compounds present. The results are therefore reliant on multivariate analysis to discriminate between the varying peak ratios over time and to group the ages accordingly. The advantage of this method is that it is not reliant on selecting a hydrocarbon to use as a standard; as was used by Zhu *et al*.^1^.

Both species can be aged to a similar time period, with week 1 clustering by itself, followed by another cluster of weeks 2 to 5. The cluster times then differ slightly between the two species. Although this last group hold a much wider time frame it is still very useful to be able to determine young puparial cases from older ones which are four months or older. Also, with the aid of other entomological evidence that maybe present (beetles, moths etc.) and the state of decomposition of the carrion, all this information could be brought together to give a more accurate age of time since colonization. Cuticular Hydrocarbons analysis can be a very good and common tool in the identification^22, 26–35^ and ageing of insects and of all life stages, from eggs to pupae to adults^1, 2, 4, 20, 21, 36–39^. This study has shown the ability of hydrocarbon analysis to age an insect specimen that can currently not be aged using any other means, opening up great potential in the field of forensic entomology.

Whilst the weathering effect on the cases is an extremely important factor, it is important to note that the changes within the hydrocarbon profiles is far less than the differences between the two species investigated in this study, and hence species identfication is still possibe, despite the effects of weathering. It is also important to note that the results presented here were obtained from standardised laboratory conditions but future work would examine puparial cases that have been exposed to the outdoor environment to study the stability of hydrocarbons and to determine what effect weathering may have on them and why these changes within the chemical profiles are being observed. This study will also be repeated using a single puparial case (rather than 2 cases of the same age) as this would be more realistic when an entomologist is sampling from a crime scene.

The main aim of this study was to preliminary investigate the potential of cuticular hydrocarbons to establish the age of empty puparial cases over a period of 9 months for two forensically important blowfly species. In summary, the results presented in this paper clearly show great potential to utilise cuticular hydrocarbons and statistical analysis to aid empty puparial case identification when currently there is no other means of doing so. Young and old cases ranging from 1 week to 9 month old ages for both forensically important blowfly species can be aged which is extremely advantageous for scenes when no other entomological evidence is present.

## Methods

### Insect materials

The colony of *L*. *sericata* used for this study was kindly supplied by the Natural History Museum in London (geographical origin, Hayward’s Heath, West Sussex, UK, 51°00′18″N:00°05′09E″). The *C*. *vicina* colony were kindly supplied by Scott Hayward’s research group (geographical location, Birmingham University campus). The flies were reared in the laboratory and maintained in a rearing cage under standard environmental conditions (23 ± 1 °C with RH ~70%) with a 14:10 h light cycle. They were supplied with sugar, water and milk powder. Pig’s liver (or pork chop for the later experiments) was used as an oviposition medium which was placed on a petri-dish with damp cotton wool to prevent the meat from drying out. Once the eggs were laid they were separated out randomly to approximately 300 eggs and placed into individual plastic containers. Eggs from one egg cluster were divided and mixed with another egg cluster to prevent effects related to the oviposition event. Once hatched, the larvae were fed daily with minced beef (approximately 50 g) and were kept in an incubator set at a temperature of 22 ± 1 °C. Resulting pupae were kept in individual containers, and the resulting puparial cases were stored at 23 ± 1 °C with RH ~60% after adult emergence.

### Sample preparation

For each species, ten replicates (*n* = *10*) were analysed, using two puparial cases per replicate. The cases were added to a 2 mL GC vial and submerged with hexane (350 μL) for 10–15 minutes. The hexane extract was collected in a clean 2 mL vial and the hexane was left to evaporate until the extract could be transferred to a 300 μL flat bottomed insert and left to dry down completely. All samples were stored dry in the refrigerator at 4 °C until they were required for analysis. The dried extract was then reconstituted in 30 μL of hexane before GC-MS analysis, which was carried out using the autosampler. A true blank and a hexane blank was run after every 10 samples (after every age cohort) to ensure no carry over in the column.

Puparial cases were extracted weekly for the first 8 weeks then monthly until 9 months. They were stored in open containers in the laboratory environment (23 ± 1 °C with RH ~60%) with a 14:10 h light cycle. Week 1 is on the seventh day once the adult fly had emerged from the puparial case, with week 2 on the 14^th^ day etc.

### Chemical Analysis: Gas Chromatography – Mass Spectrometry

Chemical analysis of all extracts was carried out on an Agilent Technologies 6890 N Network GC with a split/splitless injector at 250 °C, a Restek Rxi-1MS capillary column (30 m × 0.25 mm ID, 0.25 μm film thickness) and coupled to an Agilent 5973 Network Mass Selective Detector. The GC was coupled to a computer and data processed with Agilent Chemstation software. Elution was carried out with helium at 1 mL/min. The oven temperature was programmed to be held at 50 °C for 2 minutes then ramped to 200 °C at 25 °C/min, then from 200 °C to 260 °C at 3 °C/min and finally from 260 °C to 320 °C at 20 °C/min where it was held for 2 minutes. The mass spectrometer was operated in Electron Ionisation mode at 70 eV, scanning from 40–500 amu at 1.5 scans s^−1^. Hydrocarbons were identified using a library search (NIST08), the diagnostic fragmented ions and the Kovats indices.

### Statistical analysis

Chromatographic peak areas of *n*-alkanes, alkenes and methyl branched alkane compounds extracted from *L*. *sericata* and *C*. *vicina* were used for statistical analyses. Peak area relative abundance was arc-sine square root transformed prior to subsequent multivariate analyses. Analyses were performed using the vegan 2.0–9 library in the R statistical package^40, 41^. Following a similar approach to Pechal *et al*. 2014^36^, the Bray-Curtis distance was used for ordinations using non-metric multidimensional scaling analysis (NMDS), which is a non-parametric technique that avoids assuming linearity among community variables^42^. Additionally, permutational multivariate analysis of variance (PERMANOVA), which is a non-parametric technique based on a Bray-Curtis dissimilarity matrix^41^, tested for adult hydrocarbon profile differences among puparial case age (days/months) using the adonis function. Finally, random forest models were constructed using the randomForest 4.6–6 library in the R statistical package^40^ to identify the most important variables (e.g., specific hydrocarbon compounds) that contributed most to variation in puparial case age.
